# Dynamic stressor regimes drive shifts in biofilm-associated parasites

**DOI:** 10.1007/s00436-026-08669-3

**Published:** 2026-04-17

**Authors:** Annemie Doliwa, Ntambwe Albert Serge Mayombo, Iris Madge Pimentel, Philipp M. Rehsen, Anna-Maria Vermiert, Willem Kaijser, Lisa Voskuhl, Matthijs Vos, Bernd Sures, Micah Dunthorn

**Affiliations:** 1 Aquatic Ecology, Faculty of Biology, University of Duisburg-Essen, Essen, Germany; 2 Centre for Water and Environmental Research (ZWU), University of Duisburg-Essen, Essen, Germany; 3 Phycology, Faculty of Biology, University of Duisburg-Essen, Essen, Germany; 4 Laboratoire Interdisciplinaire des Environnements Continentaux, UMR CNRS 7360, Université de Lorraine, Avenue du Général Delestraint, Metz, 57070 France; 5 Aquatic Ecosystem Research, Faculty of Biology, University of Duisburg-Essen, Essen, Germany; 6 Department of Animal Ecology, Evolution and Biodiversity, Faculty of Biology and Biotechnology, Ruhr University Bochum, Bochum, Germany; 7 Environmental Microbiology and Biotechnology, Microbiology of Ecotones, University of Duisburg-Essen, Essen, Germany; 8 Theoretical and Applied Biodiversity Research, Faculty of Biology and Biotechnology, Ruhr University Bochum, Bochum, Germany; 9 Research Center One Health Ruhr, Research Alliance Ruhr, University of Duisburg-Essen, Essen, Germany; 10 Natural History Museum, University of Oslo, Oslo, Norway

**Keywords:** Biodiversity, Flow velocity, Freshwater, Pathogens, Salinity, Temperature

## Abstract

Parasites have gained increased recognition for their ecological relevance in riverine systems, which are highly vulnerable to biodiversity losses and degradation resulting from climate change and anthropogenic pressures. In recent decades, restoration efforts have been launched in many degraded watercourses to mitigate the influence of stressors. Yet, the ecological consequences of multiple stressor exposure and release remain complex, with the return to near-natural biotic communities representing only one possible trajectory. Parasites are still poorly understood in this context. To address this gap, we used the streamside mesocosm system *ExStream* to investigate the effects of multiple potential stressors and their release on biofilm-associated micro-eukaryotic parasites at a recently restored site of the Boye River. The Boye River system, a tributary of the Emscher River located in the Ruhr metropolis in western Germany, suffered from long-term hydromorphological degradation and pollution in the past until its restoration. In the *ExStream* experiment, we used a full-factorial design to manipulate flow velocity, salinity, and temperature during a stressor phase. The system entered a recovery phase afterwards, where the treatments were set to control conditions in all recovering mesocosms. Assessing metabarcoding data from biofilms that grew on the mesocosm walls revealed shifts in parasite assemblages between stressor and recovery phases, and reduced flow velocity was associated with altered community compositions and diversity decline. Our findings highlight that biofilm-associated parasite communities can provide important insights into the ecological dynamics of degraded and recovering river systems, and demonstrate that stressor legacies may persist beyond the period of exposure.

**Supplementary Information:**

The online version contains supplementary material available at 10.1007/s00436-026-08669-3.

## Introduction

Freshwater ecosystems are highly vulnerable to biodiversity loss and degradation driven by global climate change and anthropogenic pressures (Tickner et al. 2020). Restoration efforts of severely damaged freshwater ecosystems have been launched in recent decades, with the aim of mitigating the influence of stressors (e.g., Perini 2017). Predicting the resulting biotic communities is challenging, since recovery may take many forms, ranging from a full return to the original state to only partial or even failed recovery (Vos et al. 2023). The mechanisms underlying environmental degradation and recovery processes are complex, and dealing with multiple stressors is not straightforward due to possible stressor interactions, which may influence the direct or indirect effects on different community members (Vos et al. 2023). The diversity of aquatic organism groups of which multiple-stressor responses have been assessed so far is broad, ranging from microbial taxa to plants and metazoans (see Vos et al. 2023; Kaijser et al. 2025). However, parasites remain a poorly understood group in degraded and recovering river systems, despite their important roles in ecosystem functioning (Hudson et al. 2006; Sures et al. 2017) and their vulnerability to species loss under environmental change (Adlard et al. 2015; Wood et al. 2023; Sures et al. 2023).

Parasites add another layer of complexity to our understanding of the effects of stressors. On the one hand, parasites can modulate their hosts’ responses to and interactions with stressors (Grabner and Sures 2019; Sures and Nachev 2022). On the other hand, parasites, just like their hosts, can also be affected by stressors. Large parasites like helminths have even been used to assess pollution or environmental quality in aquatic ecosystems (Blanar et al. 2009; Sures et al. 2025). Micro-eukaryotic parasites seem less frequently studied in this respect, although they likely respond quickly to environmental changes due to their size just like other small eukaryotes (e.g., Simon et al. 2015; Gilbert et al. 2025). Furthermore, the life strategies of micro-eukaryotes are as multifaceted as those of larger parasites, which is crucial for understanding a parasite’s fate in a changing world, as it is likely linked to life cycle complexity and site of infection (Sures et al. 2023). This implies that the life cycle completion in a degraded environment is compromised by each required host, and that ectoparasites face stressors both indirectly, through their host, and directly, due to the lack of shelter provided by them (Sures et al. 2023 and references therein). Assessing stressor responses of parasites should therefore consider this variety of parasite traits and consequently, a variety of different parasitic taxa. There are many potential and widespread micro-eukaryotic parasite groups for such studies. Apicomplexa, for instance, are a highly diverse group of endoparasitic protists with different forms of complex life cycles (Votýpka et al. 2017), Chytridiomycetes and Oomycota include pathogens that produce zoospores to infect algae, animals and plants (Beakes and Thines 2016; Powell 2017), and Trypanosomatida parasitize a broad host range including humans, thereby relying on either one or two hosts (Gibson 2017). So far, the responses of several aquatic micro-parasites to a variety of different stressors such as eutrophication, flow reduction, polycyclic aromatic hydrocarbons, pulp and paper effluents or salinization have been studied (Blanar et al. 2009; Madge Pimentel et al. 2024). However, the consequences of stressor release are rarely considered. Thus, micro- or meso-cosm experiments under near-natural conditions that allow direct manipulations are particularly promising to address this gap (Sures et al. 2023), as phases of degradation and subsequent recovery could be mimicked in these systems.

Here, we employed the streamside experimental setup *ExStream*, a mesocosm system designed for simulating stream conditions (Piggott et al. 2015). The *ExStream* setup constantly supplies 64 mesocosms with water from a nearby stream, allowing colonization by small organisms that are transported into the mesocosms with stream water. While mimicking stream conditions, each of these mesocosms can be manipulated to apply treatments in a full-factorial design for testing every possible combination of stressor levels. *ExStream* experiments have been recently used to learn more about the biotic communities of the Boye River in western Germany. The Boye River is located in the Ruhr metropolis and, due to this region’s long history of coal mining and steel industry as well as a dense urbanization, most of this stream network suffered from morphological degradation and sewage discharges for decades until sectional restoration started in 1993 (Winking et al. 2014; Gillmann et al. 2023). Using *ExStream*, Madge Pimentel et al. (2024) studied responses towards salinization and reduced flow velocity across several organism groups originating from the Boye River. They found that salt had less impact, possibly due to legacy effects, while reduced water flow changed algae and macroinvertebrate communities and increased parasite diversity, among others. In the following year, another *ExStream* experiment at the same site assessed the consequences of increased salinity, increased temperature and reduced flow velocity. This setup was additionally led through a subsequent recovery phase by setting all treatments to control levels. So far, this experiment has resulted in studies on the responses of microbial decomposers (David et al. 2024), sediment microbiomes (Stach et al. 2025), and microphytobenthic communities (Mayombo et al. 2024) to stressor exposure and subsequent release. Although Mayombo et al. (2024) focused on a subset of their data which contained only eukaryotic microalgae, their full metabarcoding dataset (containing sequences amplified using general primers for the V9 hypervariable region of the small subunit ribosomal RNA) also included a number of other micro-eukaryotic groups, such as protistan and animal parasites, that they did not analyse.

Using the metabarcoding dataset from Mayombo et al. (2024), we derived and analyzed relevant parasite taxa detected in biofilms sampled after the stressor and recovery phase of *ExStream*. With the parasite subset, we asked: Did the diversity metrics and compositions of parasite communities differ (1) between degraded and recovering systems, and (2) between flow velocity, salinity and temperature treatments? Changes in the presence, abundance and diversity may signal environmental stress or rehabilitation success. Our results can therefore contribute to a better understanding of the sensitivity of micro-eukaryotic parasites associated with biofilms in degraded and recovering riverine environments. Recognizing the role of micro-eukaryotic parasites may lead to more ecologically informed river restoration approaches.

## Materials and methods

Sampling, data collection and bioinformatic processing of sequencing data were originally conducted by Mayombo et al. (2024). Below, we briefly summarize their methods and the experimental setup. Additional downstream analyses and data exploration performed in this study are described in detail afterwards.

### Experimental design and biofilm sampling

We ran the *ExStream* system in spring 2022 with water supplied from the Boye River (51.5533°N, 6.9485°E; North Rhine-Westphalia, Germany; Mayombo et al. 2024). After 20 days of acclimation, the *ExStream* setup entered a 2-week stressor phase (24 March – 7 April), and a subsequent 2-week recovery phase (7 April – 21 April). During the stressor phase, the 64 mesocosms were exposed to three potential stressors in a full-factorial design, with eight replicates for each treatment combination: flow velocity, “normal” (20 cm/s) vs. “reduced” (10 cm/s); salinity, “background” vs. “salt” (+ 200 mg/L chloride); temperature, “normal” vs. “increased” (+ 4 °C). The achieved mean parameters for flow velocity, salinity, and temperature during the stressor period are provided in Table 1 (as reported in Mayombo et al. 2024). At the end of the stressor phase, four of the eight replicates for each treatment combination, or a total of 32 mesocosms, were sampled. The remaining 32 mesocosms then entered the subsequent recovery phase, where they ran with control conditions for all stressors until they were sampled at the end of the 2-week recovery period.

**Table 1:** Achieved mean parameters for flow velocity, salinity, and temperature during the stressor phase, as well as the number of measured mesocosms (n). These values were published in the study of Mayombo et al. (2024)

	Flow velocity [cm/s]	Salinity [mS/cm]	Temperature [°C]
*Control*	14.25 ± 7.59 (*n* = 4)	0.842 ± 0.006 (*n* = 32)	8.71 ± 0.06 (*n* = 4)
*Treatment*	3.50 ± 3.32 (*n* = 4)	1.343 ± 0.151 (*n* = 32)	12.16 ± 0.08 (*n* = 4)

It is noted that, due to one mixed-up salinity application, the replicate numbers varied for four combinations in the stressor phase (see mesocosms C07 and C08 in Table S1). Specifically, there were five replicates for mesocosms treated with slow flow and increased temperature, as well as for those treated with increased salinity and increased temperature. In contrast, three replicates were available for mesocosms treated with increased temperature only, as well as for those to which all three stressors were applied. Due to technical problems or unsuitable weather conditions, the temperature treatments could be applied on nine instead of 14 days, and salinity treatments on 11 instead of 14 days during the stressor phase (David et al. 2024; Mayombo et al. 2024). More detailed information regarding the experimental setup and maintenance, treatment application and monitoring of water parameters are provided in the studies of Mayombo et al. (2024) and David et al. (2024).

Biofilms that grew on the mesocosm walls were sampled separately by Mayombo et al. (2024) at the end of the stressor (*n* = 32) and the recovery phase (*n* = 32). In brief, the stressor and recovery samples were scraped from an area of ca. 5 cm × 20 cm using plastic razor blades on the approximate opposite side of the water inflow, fixed in 96% ethanol, and partially used for metabarcoding (Mayombo et al. 2024). Additionally, in order to analyze *de novo* recolonization, an area of 2 cm × 2 cm in recovery mesocosms was freed from biofilm before entering the recovery phase, and sampled separately after the 2-week recovery period as well (*n* = 32)(Mayombo et al. 2024). The resulting two sample types of the recovery phase are here referred to as “recovery” and “recolonization”. It is noted that the mentioned differences in the sample area sizes should be considered when interpreting our results, as this could have had an impact on the diversity metrics.

### Sequencing and bioinformatic processing

Here, we only outline the major steps of the metabarcoding and bioinformatics approaches; a detailed presentation of the workflow and all programs used is published in Mayombo et al. (2024). In brief, sample preparation and DNA extraction using silica beads followed Buchner (2022a, b). Each extracted sample was amplified for the V9 hypervariable region of the small subunit ribosomal RNA genes (~ 130 base pairs) using primers 1389 F (5′-TTGTACACACCGCCC-3′) and 1510R (5′-CCTTCYGCAGGTTCACCTAC-3′) (Amaral-Zettler et al. 2009) following Stoeck et al. (2010). Libraries were sequenced at CeGaT GmbH (Tübingen, Germany) with the Illumina MiSeq system. Mayombo et al. (2024) conducted the bioinformatic analyses on the sequence data using the Natrix2 workflow (Welzel et al. 2020; Deep et al. 2023). Paired-end reads were assembled in PANDAseq v.2.11 (Masella et al. 2012), then trimmed and filtered with a threshold score of 0.9, minimum length of 77 and maximum length of 196 nucleotides. Sequences were dereplicated using the CD-HIT algorithm at 100% similarity (v.4.8.1, Fu et al. 2012). Chimeric sequences were identified and removed with VSEARCH v.2.15.2 (Rognes et al. 2016). The split-sample approach of AmpliconDuo v.1.1 (Lange et al. 2015) was applied to reduce erroneous sequences without strict abundance cut-offs. OTUs were inferred using SWARM v.3.0.0 (Mahé et al. 2022), and taxonomically assigned with the protist ribosomal reference (PR^2^) database v.4.14.0 (Guillou et al. 2013). Mayombo et al. (2024) merged the sample replicates into one representative sample by calculating the sum of reads for each OTU, from which the negative controls were then subtracted. The final OTU table that we received and filtered for relevant parasite taxa included only taxonomies assigned with a bootstrapping rate of at least 60% at and below kingdom level.

### Analyses of parasite communities

We explored the OTU table and created the figures in R v.4.3.3 (R Core Team 2024) implemented in RStudio v.2023.09.0 + 463 (Posit Team 2023), using the R-packages DHARMa v.0.4.6 (Hartig 2016), emmeans v.1.10.2 (Lenth 2017), ggpubr v.0.6.0 (Kassambara 2023), glmmTMB v.1.1.8 (Brooks et al. 2017), microViz v.0.12.4 (Barnett et al. 2021), RColorBrewer v.1.1-3 (Neuwirth 2022), readxl v.1.4.3 (Wickham and Bryan 2023), phyloseq v.1.46.0 (McMurdie and Holmes 2013), tidyverse v.2.0.0 (Wickham et al. 2019), and vegan v.2.6-4 (Oksanen et al. 2022). For the parasite OTU table (Table S2), we filtered for parasitic taxa that are known from aquatic environments (Table S3). We did not remove OTUs below a specific read number in the resulting OTU table to avoid losing rare parasites just because they were not abundant by the time of sampling.

For the α-diversity, we calculated the richness (i.e., number of observed OTUs) and Shannon diversity index which gives greater weight to rare taxa and may therefore also indicate subtle community shifts. To assess ß-diversity, we computed the Aitchison distance, and performed non-metric multidimensional scaling (NMDS). The NMDS was performed with three dimensions to achieve a stress value < 0.2. To assess the associations between the different diversity indices and experimental conditions, we used generalized linear models (GLMs) with the independent variables phase, flow velocity, salinity and temperature. The residuals of the resulting GLMs were checked with the DHARMa package. For richness, we chose the negative binomial distribution (quadratic parameterization, nbinom2) with the log link function, and for the Shannon index, the Gamma distribution with the identity link function. With the betadisper() function, we calculated the beta dispersion grouped by phases from the Aitchison distance matrix to use as a response variable for ß-diversity, which is expressed as the Euclidean distance from the centroid. We chose the Gaussian distribution and identity link function. We estimated marginal means (Emmeans) based on these GLMs and compared them pairwise using the pwpm() function, which also included tukey adjustment by default.

For a better understanding of community shifts, we looked for changes within top taxa and in the relative read abundances of specific parasite taxa. For the analysis of the top taxa, we selected only the top 10 most abundant OTUs of each individual sample and visually compared the compositions. For the analysis of the relative read abundances of specific taxa, we selected micro-eukaryotic taxa that, to our knowledge, are considered obligate parasites (Apicomplexa, Ichthyobodonidae, Ophryoglenida, Perkinsea, Phytomyxea, and Trypanosomatida), or that were represented by high OTU counts in our data set (Chytridiomycotina, Labyrinthulomycetes, Oomycota, and Vampyrellida; Table S3). We agglomerated the relative abundances at the taxonomic levels mentioned in brackets to use as response variables in GLMs with the beta distribution and logit link function, again followed by calculating and comparing Emmeans. For the taxa Ichthyobodonidae, Ophryoglenida and Perkinsea, we used a zero-inflated component in the respective GLMs because the beta distribution is defined only on the open interval (0, 1) and therefore cannot accommodate observations equal to zero.

## Results

### Occurrence of parasites in biofilms

We selected 31 parasitic taxa (Table S3), resulting in a filtered OTU table with 681,240 reads and 860 OTUs. These represent 2.18% of a total of 31,225,131 reads and 8.83% of a total of 9,741 OTUs in the provided OTU table. Most OTUs belonged to the Apicomplexa (198 OTUs, or 23.02% of all OTUs in the filtered OTU table), Oomycota (160 OTUs, 18.60%), Labyrinthulomycetes (135 OTUs, 15.70%), and Chytridiomycotina (109 OTUs, 12.67%; Table S3). More generally, the dominating supergroups in the filtered OTU table were the Stramenopiles (297 OTUs, 34.53%), followed by the Alveolata (238 OTUs, 27.67%), and Opisthokonta (161 OTUs, 18.72%). The remaining OTUs belonged to the Rhizaria (84 OTUs, 9.77%), Excavata (72 OTUs, 8.37%), and Amoebozoa (8 OTUs, 0.93%). Among the Opisthokonta were also 26 metazoan parasite OTUs, including cestodes, a leech, monogeneans, myxozoans, nematodes, a nematomorph, and trematodes (Table S3).

### Alpha diversity analyses

**Fig. 1:**
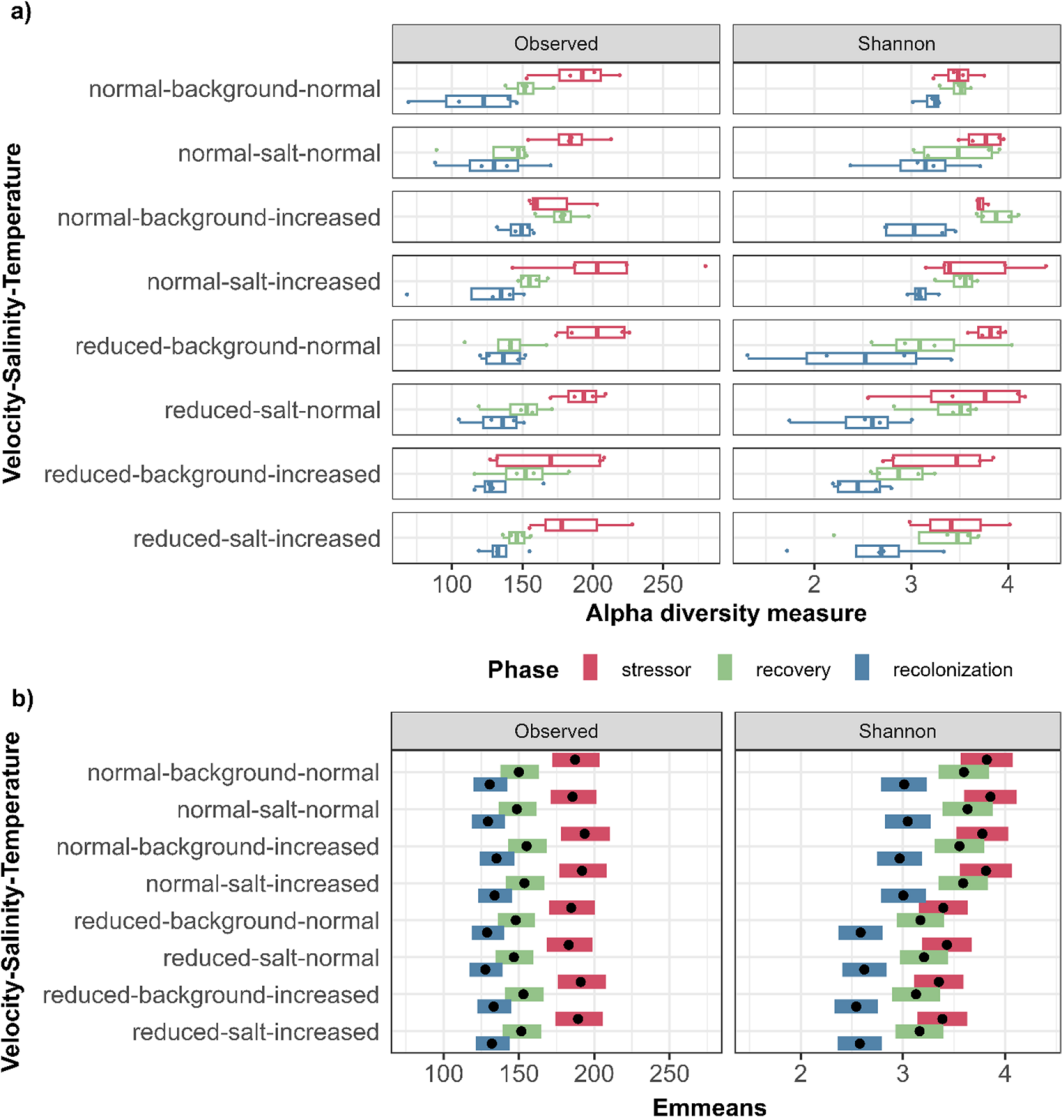
(**a**) The observed number of OTUs and Shannon index, according to stressor treatment combinations applied during the stressor phase, and (**b**) the estimated marginal means (Emmeans) for the same alpha diversity indices

OTU counts per sample varied between 68 and 280 OTUs (mean ± standard deviation: 156.8 ± 35.45 OTUs, Figs. 1 and S1), and the mean tended to be the highest after the stressor phase (188.19 ± 32.43 OTUs, Table S4), and the lowest in recolonized biofilms (131.38 ± 24.44 OTUs), with recovery samples being in between (150.91 ± 21.95 OTUs). For the OTU count, we found a significant negative relation with the recovery and recolonization phases, but not with any stressor treatment (see Table 2). We identified the same pattern for the respective estimated mean OTU count, as it was highest in the stressor phase, which significantly differed from recovery and recolonization samples within the same treatment group (*p* < 0.001, Fig. 1b; Table S5). Noteworthy, this trend was also found for the control mesocosms.

**Table 2:** Generalized linear model (GLM) calculated from the observed number of OTUs and Shannon diversity index. Values rounded to three decimal places. Significances coded as 0.1 ‘.’, 0.05 ‘*’, 0.01 ‘**’, 0.001 ‘***’

		Estimate	Std. Error	Z value	Pr(>|z|)	
Observed	(Intercept)	5.232	0.043	122.676	<0.001	***
	Phase recovery	-0.222	0.042	-5.244	<0.001	***
	Phase recolonization	-0.360	0.043	-8.433	<0.001	***
	Velocity reduced	-0.014	0.035	-0.392	0.695	
	Salinity salt	-0.009	0.035	-0.259	0.796	
	Temperature increased	0.033	0.035	0.958	0.338	
Shannon	(Intercept)	3.820	0.130	29.428	<0.001	***
	Phase recovery	-0.224	0.129	-1.731	0.083	.
	Phase recolonization	-0.808	0.119	-6.775	<0.001	***
	Velocity reduced	-0.425	0.098	-4.358	<0.001	***
	Salinity salt	0.035	0.097	0.367	0.714	
	Temperature increased	-0.044	0.097	-0.451	0.652	

The Shannon index varied between 1.31 and 4.39 (mean ± standard deviation: 3.26 ± 0.58, Fig. 1 and S1). Just like the OTU count, the diversity was on average the highest after the stressor phase (3.59 ± 0.42, Table S4), the lowest in the recolonized samples (2.81 ± 0.56), and in between after the recovery phase (3.38 ± 0.46). The mean Shannon index was notably higher in recovery and recolonization samples that received normal flow velocity (3.59 ± 0.30 and 3.11 ± 0.32, respectively) compared to those with a previous reduction (3.17 ± 0.50 and 2.50 ± 0.58). In contrast, the differences according to salinity or temperature treatments appeared generally less pronounced (Fig. 1 and S1; Table S4). We found a significant negative relation between the Shannon index and recolonization as well as flow velocity (see Table 2). Treatment groups that only varied in the applied flow velocity had significantly different estimated mean diversities from each other, as slow flow was associated with a lower estimated Shannon index (*p* < 0.01 each, Fig. 1b, Table S5b). Furthermore, stressor and recovery samples of the same treatment group both significantly differed from recolonized samples in that they were comparably more diverse (*p* < 0.001 each), but not from each other (*p* > 0.05 each, Fig. 1b, Table S5b).

It is further noteworthy that recovery samples that had been treated with the combination of normal flow velocity, background salinity and increased temperature showed a comparably high OTU count (mean ± standard deviation: 178.25 ± 15.56 OTUs) and Shannon index (3.88 ± 0.21; Fig. 1, Table S4).

### Beta diversity analyses

**Fig. 2:**
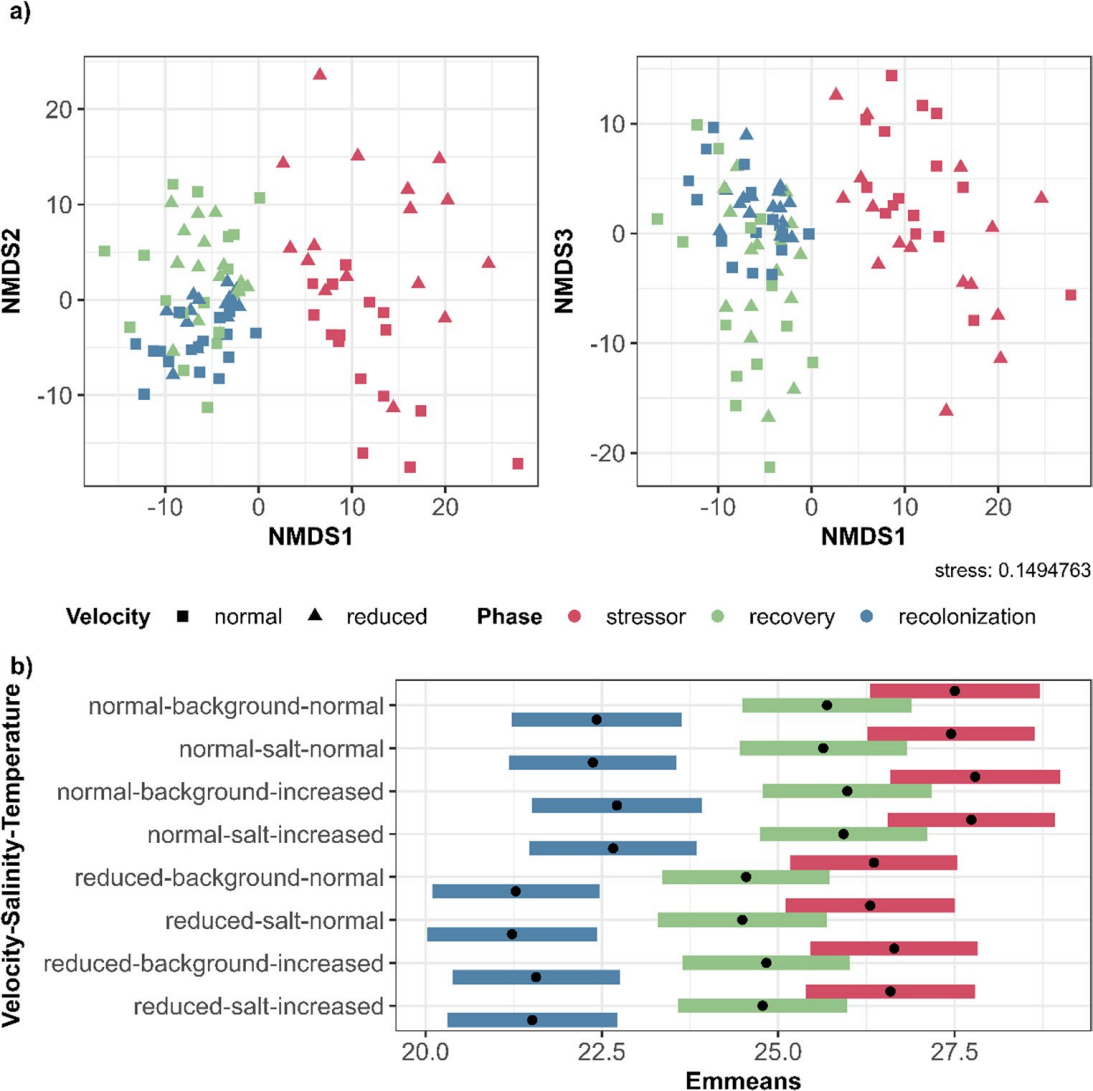
(**a**) Three-dimensional NMDS with Aitchison distances, with colors indicating the experimental phase, and the symbols indicating the flow velocity treatment. Each point represents the parasite community of one sample. (**b**) Estimated marginal means (Emmeans) of the beta dispersion, as calculated from the Aitchison distance matrix

In the NMDS plots, the parasite communities clustered according to stressor phase, recovery phase, and recolonization, with a strong overlap between the latter two (Fig. 2a). Within the clusters of stressor phase and recolonization, we visually identified a discernible pattern according to flow velocity (Fig. 2a), while no sub-clusters were standing out for salinity and temperature treatments (Fig. S2).

The beta dispersion calculated from the Aitchison distances was significantly associated with recovery and recolonization as well as with flow velocity (see Table 3). Beta dispersion was highest for the stressor phase, closely followed by recovery, indicating that compositional variance was the highest between samples from the stressor phase, while it was the lowest between recolonized biofilms (Fig. 2b). Generally, compositional variance was lower in treatment groups that included a reduced flow velocity (Fig. 2b). Pairwise comparisons revealed that the estimated beta dispersion from recolonized biofilms was significantly different from stressor and recovery samples (*p* < 0.001 each, Table S6).

**Table 3:** Generalized linear model (GLM) calculated from the beta dispersion of Aitchison distances. Values rounded to three decimal places. Significances coded as 0.1 ‘.’, 0.05 ‘*’, 0.01 ‘**’, 0.001 ‘***’

	Estimate	Std. Error	Z value	Pr(>|z|)	
(Intercept)	27.504	0.605	45.454	< 0.001	***
Phase recovery	-1.813	0.601	-3.017	0.003	**
Phase recolonization	-5.078	0.601	-8.453	< 0.001	***
Velocity reduced	-1.147	0.491	-2.336	0.019	*
Salinity salt	-0.054	0.491	-0.109	0.913	
Temperature increased	0.287	0.491	0.586	0.558	

### Composition of biofilm-associated parasite communities

We noticed compositional differences according to treatments and phases at a high taxonomic level (Fig. S3): For example, the Discoba often represented larger proportions in stressor samples with normal compared to reduced flow velocity (Fig. S3a), and most recolonized biofilm communities strongly shifted towards fungi, especially under slow flow (Fig. S3c). To explore such shifts in more taxonomic and compositional detail, we pulled out only the top 10 most read-abundant OTUs of each sample.

**Fig. 3:**
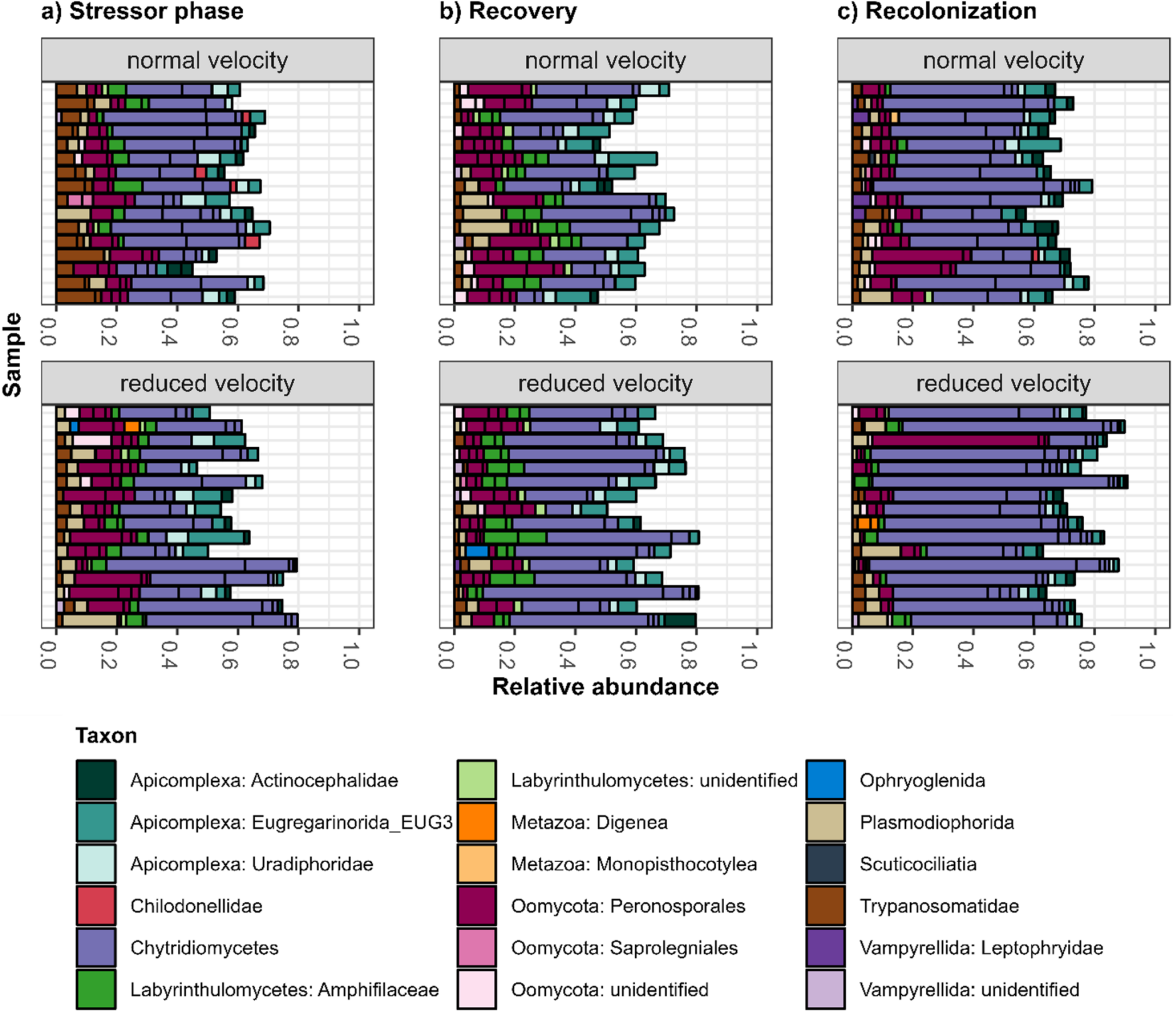
Relative abundances of the top 10 OTUs in each mesocosm after the (**a**) stressor phase, (**b**) recovery phase, and (**c**) recolonization during recovery. Facet labels indicate the flow velocity treatment. Note that the mesocosms were grouped according to the flow velocity treatment, resulting in 16 replicates for each combination of flow velocity level and phase

The top 10 OTUs made up between 45.05% and 90.88% of a sample, and 66.47% on average (Fig. 3 and S4). An OTU belonging to the Chytridiomycetes was the most abundant one in 78 out of the 96 samples (Fig. 3, Table S7), and further taxonomically assigned to the Rhyzophidiales, an order that includes obligate and facultative parasites of algae (Frenken et al. 2017) (Table S7). We found the most distinctive trends for flow velocity (Fig. 3). In stressor samples, there were shifts towards the Trypanosomatida under normal flow, whereas the Oomycota and Plasmodiophorida tended to reach higher proportions under reduced flow velocity (Fig. 3a). In recovery samples, we found shifts towards the Chytridiomycetes in mesocosms treated with slow flow, and the proportion of the Plasmodiophorida and Oomycota appeared reduced in several samples (Fig. 3b). In recolonized biofilms, the top 10 subsets strongly shifted towards the Chytridiomycetes, especially when the flow velocity was previously reduced. In contrast, the Apicomplexa and Oomycetes made up smaller proportions under reduced flow velocity (Fig. 3c). In the context of salinity treatments, we did not identify clear patterns. For temperature, the Plasmodiophorida appeared relatively more abundant among the top 10 OTUs in the recovery phase under normal temperature. In contrast, the Oomycota tended towards higher proportions in recovery and recolonization samples under increased temperature (compare Fig. S3b, c).

We selected specific parasite taxa to check whether their agglomerated relative read abundances were associated with any phases or treatments (Fig. S5, Table S8). We found significant positive associations with the recovery phase for the Labyrinthulomycetes and Vampyrellida (*p* < 0.001 and *p* < 0.05, Table S8e, j), as well as negative associations for the Endomyxa-Phytomyxea and Trypanosomatida (*p* < 0.01 and *p* < 0.001, Table S8c, i). The recolonization was positively associated with the abundances of the Chytridiomycotina (*p* < 0.001, Table S8b), and negatively associated with those of the Endomyxa-Phytomyxea (*p* < 0.01), Labyrinthulomycetes (*p* < 0.001), Oomycota (*p* < 0.05) and Trypanosomatida (*p* < 0.001; Table S8c, e, f, i). We identified a significant positive relation of reduced flow velocity with the Chytridiomycotina, and negative ones with the Apicomplexa and Trypanosomatida (*p* < 0.001 each, Table S8a, b,i). For increased temperature, we found a significant negative association with the Labyrinthulomycetes (*p* < 0.01, Table S8e), and none with increased salinity (*p* > 0.05, Table S8). The pairwise comparisons of the estimated relative mean abundances revealed some significant phase- and treatment-associated patterns (Fig. S5, Table S9). Among the most striking observations were the Trypanosomatida, for which the estimated relative read abundances were significantly higher in the stressor phase compared to the recovery and recolonization (*p* < 0.001 each), and lower under reduced flow velocity across all phases (*p* < 0.001 each, Table S9i). The Chytridiomycotina had significantly higher abundances in recolonized biofilms compared to stressor and recovery samples under every treatment combination (*p* < 0.001, Table S9b). For the Labyrinthulomycetes, no significant differences associated with temperature treatments were recovered (*p* > 0.05, Table S9d). However, they significantly varied between all three phases, with the highest estimated relative read abundances in the recovery, and the lowest in the recolonization (stressor and recovery phase: *p* < 0.01, recolonization and stressor or recovery phase: *p* < 0.001). In contrast, no such significant differences were uncovered for the Endomyxa-Phytomyxea, Ichthyobodonidae, Oomycota, Ophryoglenida, Perkinsea, and Vampyrellida (*p* > 0.05, Table S9c, e-h, j).

## Discussion

We applied the *ExStream* mesocosm system to test how reduced flow velocity, salinization, and warming influence biofilm-associated parasite communities during stressor exposure, stressor release (recovery), and recolonization. We focused on parasites associated with the biofilms that grew in the considered mesocosms. Biofilms are complex entities packed with microbial life, and have already been used as early warning systems for stressors in fluvial ecosystems (Sabater et al. 2007). In the analyzed biofilms, we identified diverse parasite taxa, along with additional taxa being likely parasitic. Several of these taxa are presumed to parasitize members of the biofilm community. For example, some Chytridiomycota, Oomycota and Labyrinthulomycota can infect micro-algae (Carney and Lane 2014), and Phytomyxea can infect brown algae, diatoms, and oomycetes (Neuhauser et al. 2011). Others inhabit biofilms, such as the free-living opportunistic amoebae genera *Acanthamoeba* and *Naegleria* (Scheid 2018), or may possibly employ them as reservoirs, as has for example been observed for the oocysts of an apicomplexan (Wingender and Flemming 2011). Some other taxa parasitize the associated fauna. For example, trematodes can infect snails and even manipulate their grazing behavior (Vivas Muñoz et al. 2018), mosquito larvae are potential hosts to oomycetes (Kaczmarek and Boguś 2021), and ichthyosporeans are parasites of various metazoans including invertebrates and fish (Shabardina et al. 2024). We would like to stress, however, that we focused on higher taxonomical levels when filtering the OTU table. Since several taxa are not exclusively parasitic, meaning that they generally comprise species of different life strategies as well, we might also have included some free-living species. Future analyses, such as the construction and exploration of co-occurrence networks (e.g., Doliwa et al. 2021), may provide a good starting point for disentangling the underlying symbiotic relationships. Given the taxa found, we nevertheless conclude that the assessed Boye River site harbors a broad micro-eukaryotic parasite spectrum with different hosts and life strategies.

Through our mesocosm approach, we found that parasite communities were more diverse during stressor phases than following a recovery phase. This pattern was similarly found for micro-algae from the same samples (Mayombo et al. 2024). We assume seasonal effects influenced the biofilms in all mesocosms of the same phase equally, as protists can respond very quickly to such changes. Simon et al. (2015), for example, observed complex and rapid temporal dynamics as well as seasonal patterns in freshwater micro-eukaryotic community structures. Biofilm aging processes could have further contributed to these general shifts. For instance, Jackson et al. (2001) described that the richness of bacterial biofilm assemblages initially increased in the first week, then slowly declined, and ultimately increased again after two to three months. The *de novo* recolonized and thus ‘youngest’ communities in our study were the least diverse and strongly shifted towards Chytridiomycotina. It is important to note that the recolonized biofilms were sampled from a smaller area than the stressor and recovery biofilms (Mayombo et al. 2024). Sample volumes can impact diversity metrics in sequencing studies, with smaller volumes potentially yielding lower numbers of unique OTUs, as has been shown for benthic metazoans and micro-eukaryotes in marine sediment samples (Nascimento et al. 2018). Other studies reported no significant effects of the sample amount on the taxonomic information, for example Kindtler et al. (2025) who studied microbial communities in rhizospheres. Due to these uncertainties, we assume that the comparability between recolonization samples and the other two sample types is most likely limited. Interpretation of the patterns observed in the recolonized biofilms should therefore focus more on their responses to treatments, and less on comparisons to other phases. The parasites may have also mirrored the spectrum of available hosts, which first have to return to the scraped biofilm area and establish in order for their parasites to do the same. Parasite diversity has previously been reported to increase with host diversity in different host-parasite systems, for example in fleas of small mammals (Krasnov et al. 2004) or amphibian-infecting parasites (Johnson et al. 2016). In addition, Mayombo et al. (2024) observed priority effects in the microphytobenthic biofilm communities; accordingly, symbionts of the detected chytrids, such as algal hosts, were likely among the first recolonizers, which may contribute to their higher relative abundances.

We identified flow velocity in all experimental phases to be the most relevant stressor that we tested in shaping the parasite communities, while elevated water temperatures and increased salinity apparently had no strong effect. Water flow poses a critical factor for host-parasite interactions, as it can for instance affect host finding or the host’s susceptibility to parasite infections (e.g., Lenihan et al. 1999; Kühn and Hofmann 1999; Wunderlich et al. 2024). The two flow velocities in our study did not lead to a significantly altered parasite richness during the stressor phase, but rather resulted in different community compositions with overall decreased diversities. An increased parasite diversity under slow flow as observed in the previous *ExStream* setup at the same Boye River site (Madge Pimentel et al. 2024) was not detectable in the stressor phase. The comparability of these two *ExStream* experiments was, however, limited. We ran the here-analyzed experiment one month earlier the following year, a time shift that is important to consider for small eukaryotes, whose communities can rapidly change even between months (e.g., Simon et al. 2015), and even more so for parasitic ones, for which seasonal variation is a long-documented phenomenon (e.g., Canter and Lund 1948). The responses to an environmental variable can strongly vary between parasites whose vulnerability depends on their respective life histories (Sures et al. 2023). Seasonal variation in the occurrence and abundance of parasites with distinct traits, or vulnerabilities, may have therefore contributed to the different outcome.

A change from reduced back to normal flow velocity led to mixed responses from parasite community members, expressed in decreased Shannon diversities with a comparable OTU richness in recovering mesocosms. Parasites of the same community responding differently to streamflow have previously been linked to life cycle traits, as reported for the mostly metazoan parasite community in the fish *Fundulus zebrinus* by Janovy et al. 1997, for example. Their findings included that trematodes were less abundant and prevalent under high water, possibly because the numbers of intermediate hosts or free-swimming infective larvae were reduced, whereas *Trichodina* sp. ciliates with their short and direct life cycles responded more dynamically (Janovy et al. 1997). We assume that in our study the affected communities may have shifted away from parasites profiting from slower flow, an environmental factor that can be beneficial for the accumulation of dispersive stages and host encounter rates (e.g., Marcogliese 2016; Wunderlich et al. 2024), or for the required hosts. Chytridiomycotina seemed robust against an active or previous treatment with slow flow. Parasitic chytrids use zoospores to actively find and penetrate their hosts, and some may switch to resting stages or to saprotrophy if no suitable host is present (Frenken et al. 2017 and references therein), which could explain their resilience in our experiment. By contrast, Apicomplexa and Trypanosomatida were relatively less abundant in association with slow flow. These two taxa are exclusive parasites whose life cycle complexities and host ranges can vary (Votýpka et al. 2017; Kaufer et al. 2017), offering several possible stressor targets, which should be explored in subsequent studies. The observations made for recolonized biofilms, which never directly experienced the stressor treatment, could be further explained by the surrounding biofilms, whose inhabitants contributed to the recolonization process and thus brought the legacy of reduced flow velocity with them. The observed priority effects for algae in these samples (Mayombo et al. 2024) are likely relevant here. Notably, as already suggested by David et al. (2024) in their assessment of aquatic hyphomycetes in this ExStream, the patterns observed in our study may, at least in part, reflect a delayed response to the flow-reduction treatment. It follows that a longer duration of the experiment could provide greater clarity about treatment effects.

Increased temperature and increased salinity did not lead to comparably strong shifts in the community compositions. Both are nonetheless environmental factors that are known to potentially affect parasites. Warming can influence host-parasite interactions, life histories, parasite community structures, and transmission dynamics, but the consequences can be complex and difficult to predict (reviewed by Sures et al. 2023). The impact of salt contamination can vary between parasites as well, ranging from reduced infection loads to increased salinity tolerance of the host to higher infection intensities (Piscart et al. 2007; Stockwell et al. 2015; Milotic et al. 2017). We only found the Labyrinthulomycetes tending to be negatively affected by increased temperatures, a taxon of especially marine protists which are found on many surfaces and that include parasites, saprotrophs, and organisms with an unclear trophic status (Pereboev and Bubnova 2023). Niche partitioning according to water temperature, among other environmental parameters, has been described in marine Labyrinthulomycetes (Xie et al. 2022), and a study on the seagrass-infecting *Labyrinthula* sp. reported that warming reduced the occurrence and severity of an infection (Olsen et al. 2015). However, more investigations on Labyrinthulomycetes in freshwater environments are needed to verify potential temperature effects. It is further noted that the applied temperature treatment may have been too short or not strong enough to pose a real stressor to the parasite taxa assessed in this study. That we identified no discernible effects of salinization on the assessed parasite communities is in overall accordance with previous findings from the Boye River (Madge Pimentel et al. 2024). The Boye River mouth in the past could reach chloride concentrations of 15,500 mg/L in 1996, until restoration efforts pushed the salinity down to concentrations like 26 mg/L as measured in 2021 (Madge Pimentel et al. 2024). Salinity-tolerant or adapted taxa are therefore probably still abundant in the Boye River, while taxa that are more sensitive are yet to return. This may also be true for the present parasites, which either benefit from their hosts’ adaptations or whose external stages, if any, are robust against higher salinities. As already found in several *ExStream* studies from the Boye River (Madge Pimentel et al. 2024; David et al. 2024; Mayombo et al. 2024), therefore, we likely observed legacy effects.

## Conclusion

Our experimental mesocosm setup allowed us to test the impact of the exposure and release of three potential stressors (reduced flow velocity, increased salinity, increased temperature) on important parasite taxa in biofilms.

Diversity generally differed between stressor and recovery phases, indicating potential biofilm aging processes and seasonality reflected by the parasite communities. While we did not identify warming and salinization as relevant stressors in our experiment, our findings point toward flow velocity likely affecting the community composition that showed a decline in diversity. A potential delayed response to a prior flow reduction may indicate a decline in adapted taxa. We conclude that the legacy of reduced flow velocity can persist, even in *de novo* recolonized samples of the recovery phase that never directly faced the stressor. A slowed-down water flow, or more generally a loss in water flow variation, may hamper a quick return to near-natural micro-eukaryotic parasite communities. Subsequent investigations can especially focus on relevant taxa such as the apicomplexans, chytrids, or trypanosomatids over longer time spans to better understand their fate in changing riverine systems.

## Supplementary Information

Below is the link to the electronic supplementary material.

Supplementary Material 1

Supplementary Material 2

## Data Availability

The original, unfiltered OTU table was provided by Mayombo et al. (2024), and their data are also available in the GitHub repository: https://github.com/sergemayombo/exstream2022. The bootstrap-filtered parasite OTU table subset analyzed here is provided in the Supplementary Information of our study (Table S2).
