# Supplementary material for: Dynamic stressor regimes drive shifts in biofilm-associated parasites
Source: Parasitol Res. 2026 Apr 17;125(1):56. doi: 10.1007/s00436-026-08669-3 (PMC13090197; doi:10.1007/s00436-026-08669-3)
Supplement: Supplementary file 1 — Supplementary Material 1 [file 436_2026_8669_MOESM1_ESM.pdf]

Supporting Information

Journal: Parasitology Research

Article title: Dynamic stressor regimes drive shifts in biofilm-associated parasites

Authors and affiliations:

Annemie Doliwa[1,2*,] Ntambwe Albert Serge Mayombo[2,3,4], Iris Madge Pimentel[2,5], Philipp M. Rehsen[2,5], Anna-Maria Vermiert[6], Willem Kaijser[1], Lisa Voskuhl[2,7], Matthijs Vos[8], Bernd Sures[1,2,9], Micah Dunthorn[10]

[1]Aquatic Ecology, Faculty of Biology, University of Duisburg-Essen, Essen, Germany
[2]Centre for Water and Environmental Research (ZWU), University of Duisburg-Essen, Essen, Germany
[3]Phycology, Faculty of Biology, University of Duisburg-Essen, Essen, Germany
[4]Laboratoire Interdisciplinaire des Environnements Continentaux, UMR CNRS 7360, Université de Lorraine, Avenue du Général Delestraint, 57070 Metz, France
[5]Aquatic Ecosystem Research, Faculty of Biology, University of Duisburg-Essen, Essen, Germany
[6]Department of Animal Ecology, Evolution and Biodiversity, Faculty of Biology and Biotechnology, Ruhr University Bochum, Bochum, Germany
[7]Environmental Microbiology and Biotechnology, Microbiology of Ecotones, University of Duisburg-Essen, Essen, Germany
[8]Theoretical and Applied Biodiversity Research, Faculty of Biology and Biotechnology, Ruhr University Bochum, Bochum, Germany
[9]Research Center One Health Ruhr, Research Alliance Ruhr, University of Duisburg-Essen, Essen, Germany
[10]Natural History Museum, University of Oslo, Oslo, Norway

***Corresponding author:** Annemie Doliwa, annemie.doliwa@uni-due.de

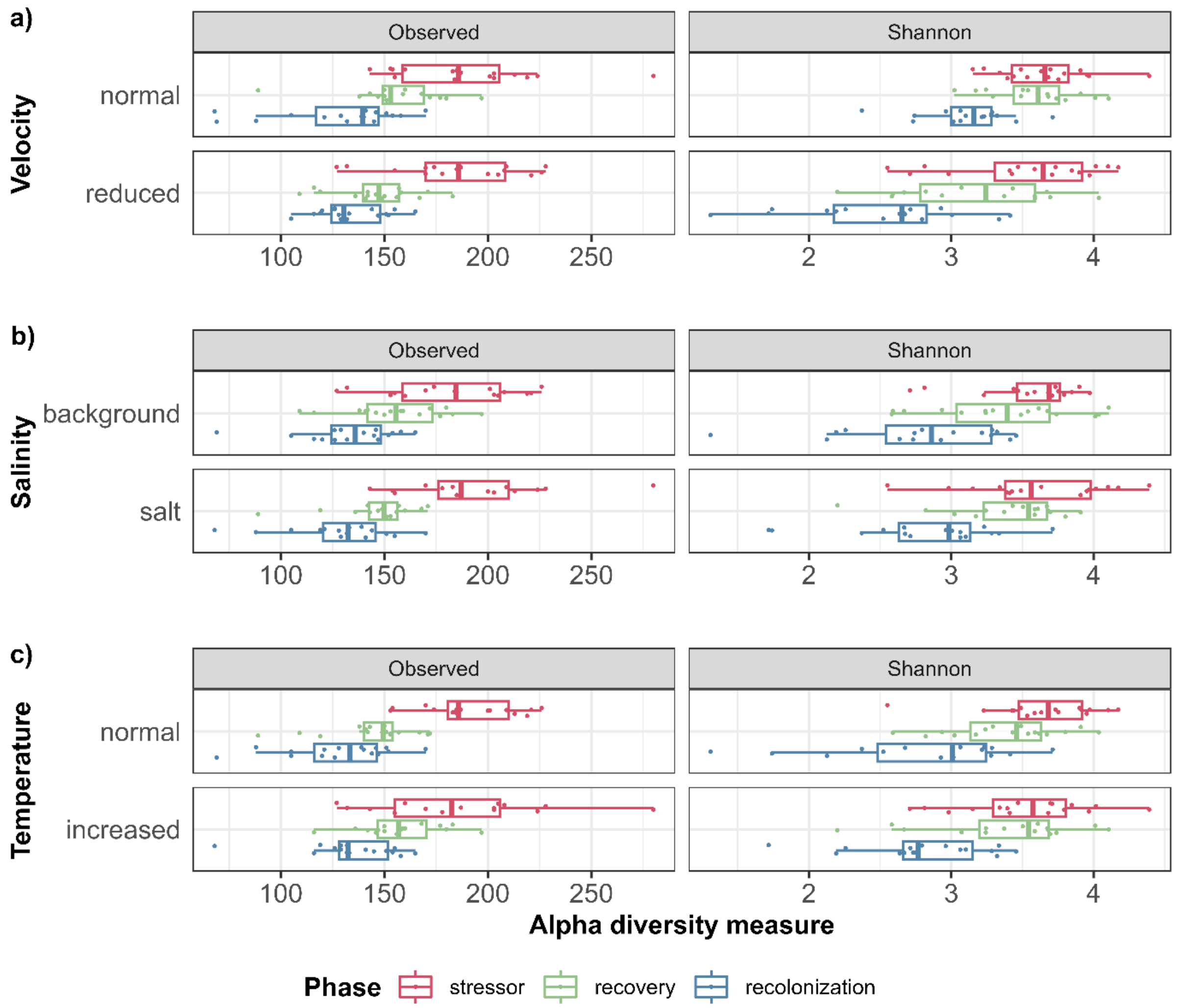

Fig. S1 Alpha diversity indices (Observed OTUs per sample, Shannon index) of micro-eukaryotic parasites in the *ExStream* system according to the single stressor treatments a) flow velocity, b) salinity, and c) temperature. Colors indicate the experimental phase.

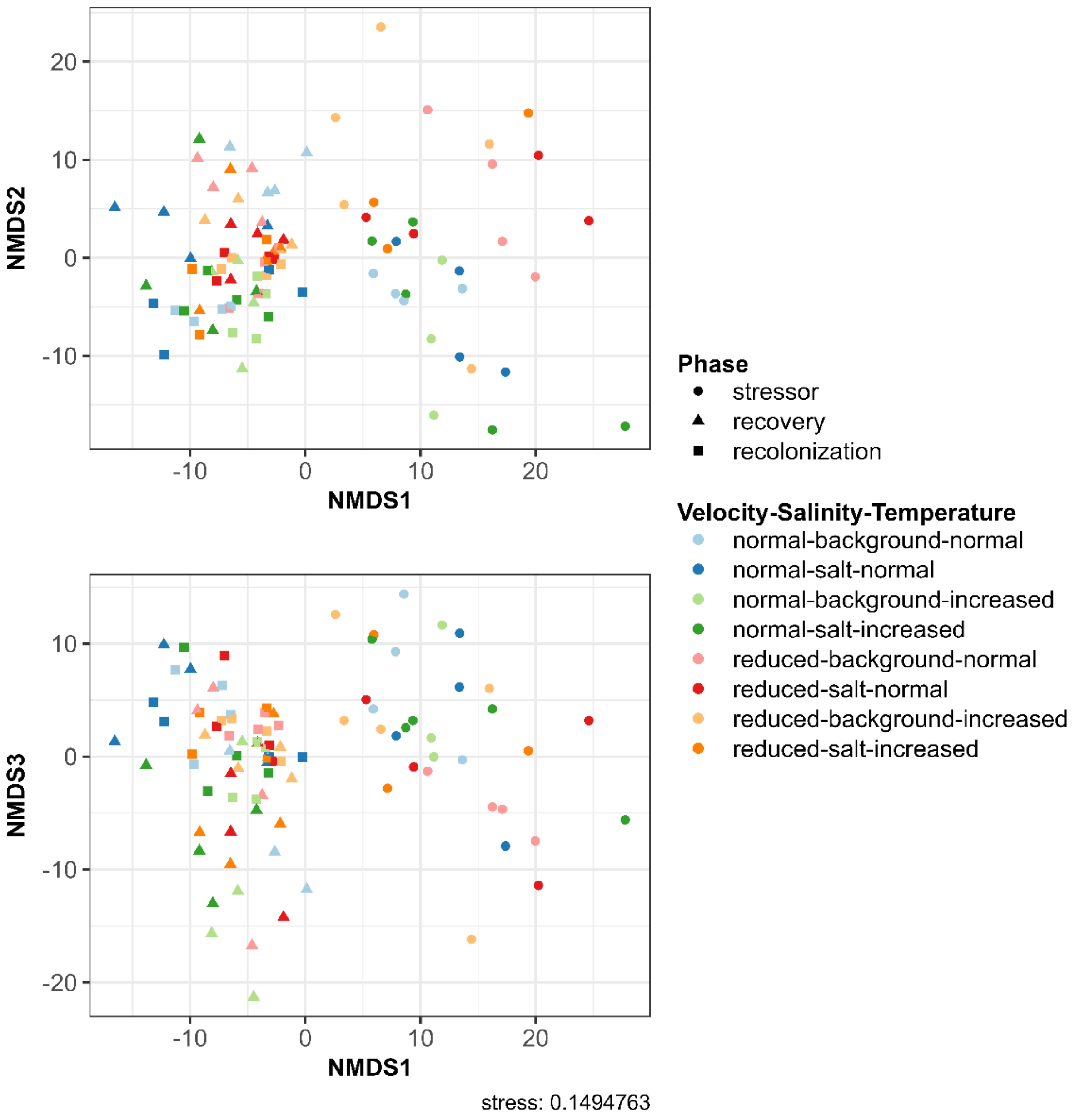

Fig. S2 Three-dimensional NMDS based on Aitchison distances as calculated for biofilm-associated parasite communities in the *ExStream* system. Colors indicate the different treatment combinations of flow velocity, salinity and temperature, and the symbols represent the experimental phase.

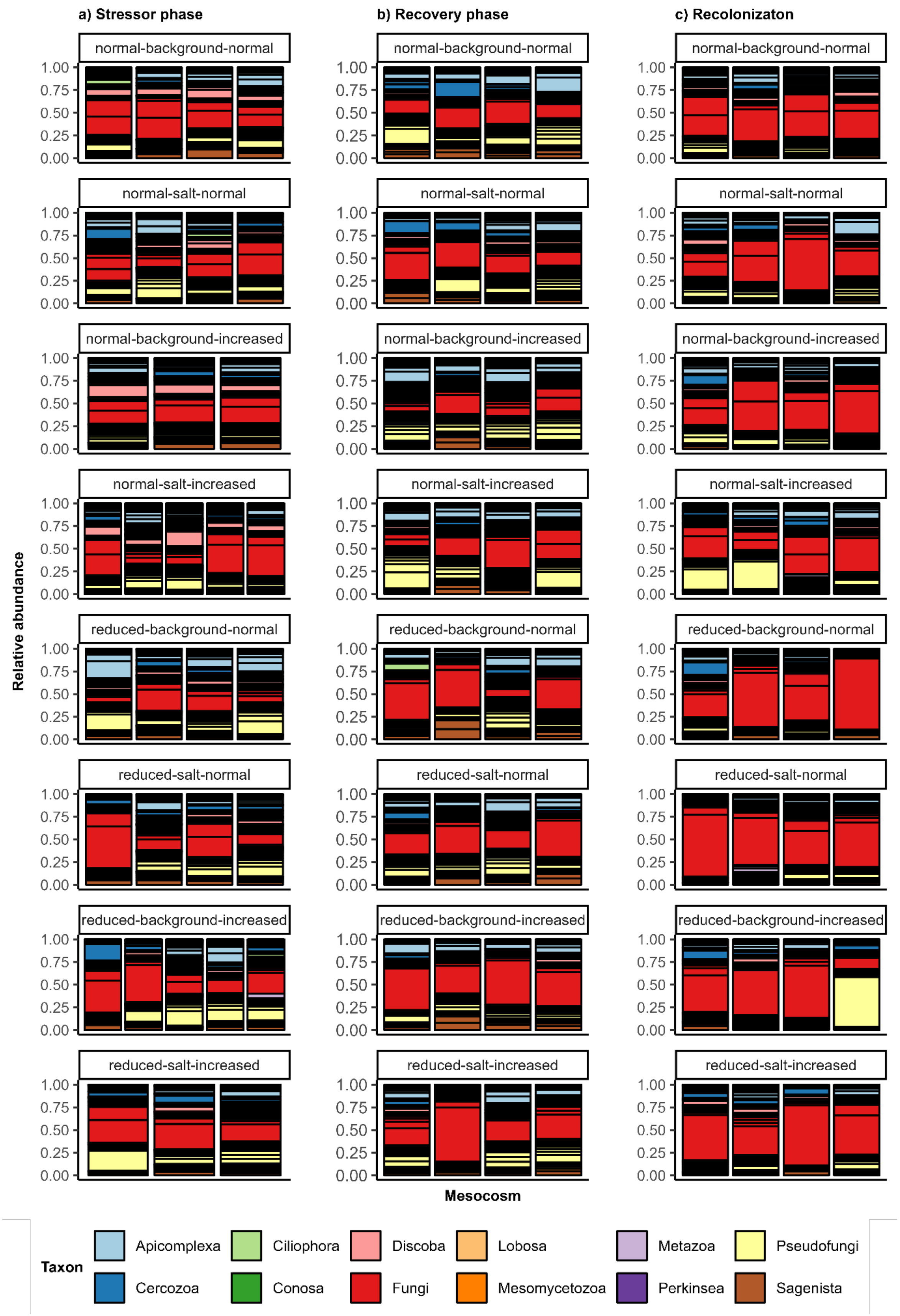

Fig. S3 Taxonomic composition of the biofilm-associated parasite communities in the *ExStream* system, according to experimental phase (a-c) and the different stressor treatment combinations (Velocity-Salinity-Temperature; see facet labels). Black lines within the columns frame individual OTUs. Colors indicate the taxonomic assignment.

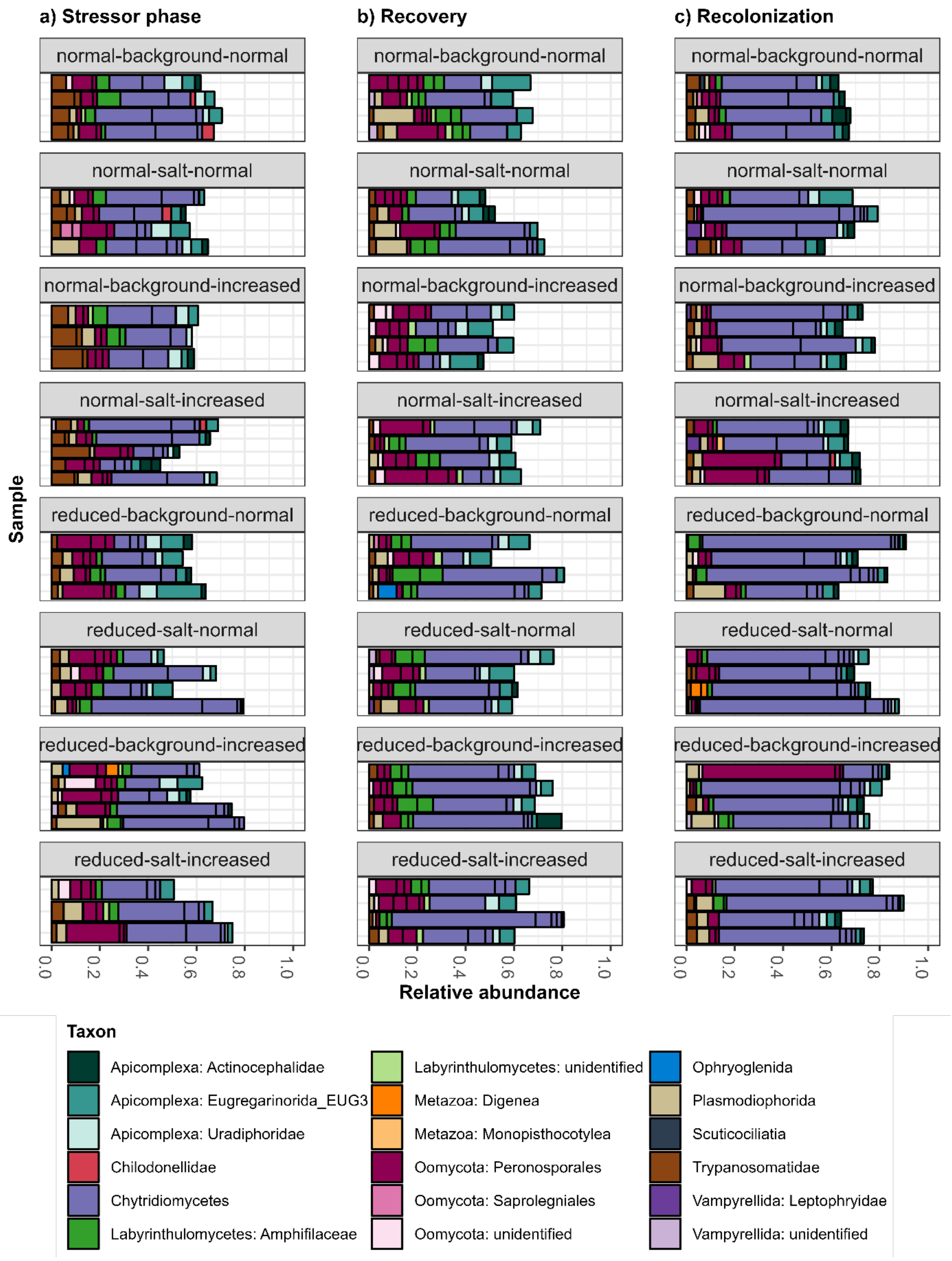

Fig. S4 Relative abundances of the top 10 OTUs in the biofilm-associated parasite communities in each mesocosm, according to the a) stressor phase, b) recovery phase, and c) recolonization of the *ExStream* experiment. Facet labels indicate the stressor treatment combination (Velocity-Salinity-Temperature). Black lines within the columns frame individual OTUs, and the colors indicate the taxonomic assignment.

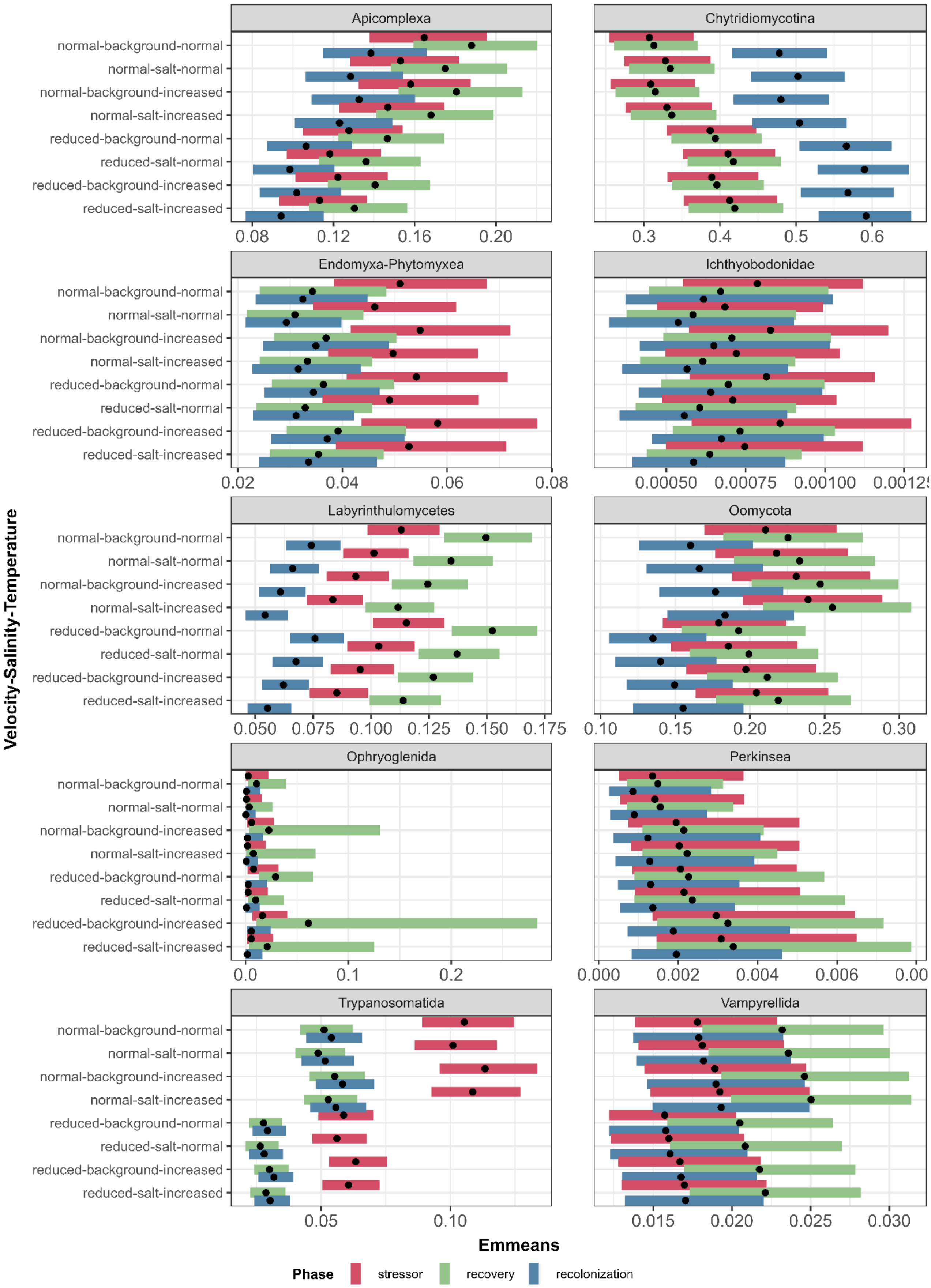

Fig. S5 Estimated marginal means (Emmeans) of the relative read abundances for each treatment combination (Velocity-Salinity-Temperature) based on the GLMs calculated for several micro-eukaryotic parasite taxa (see facet labels). Colors indicate the experimental phase of the *ExStream* experiment.
